# Pneumonia knowledge and care seeking behavior for children under-five years in Jigawa, Northwest Nigeria: a cross-sectional study

**DOI:** 10.3389/fpubh.2023.1198225

**Published:** 2023-07-18

**Authors:** Ayobami A. Bakare, Carina King, Julius Salako, Damola Bakare, Obioma C. Uchendu, Rochelle Ann Burgess, Funmilayo Shittu, Agnese Iuliano, Adamu Isah, Tahlil Ahmed, Samy Ahmar, Paula Valentine, Temitayo Folorunso Olowookere, Eric D. McCollum, Tim Colbourn, Adegoke G. Falade, Hamish R. Graham

**Affiliations:** ^1^Department of Global Public Health, Karolinska Institute, Stockholm, Sweden; ^2^Department of Community Medicine, University College Hospital, Ibadan, Nigeria; ^3^Department of Paediatrics, University of Ibadan, Ibadan, Nigeria; ^4^Department of Community Medicine, University of Ibadan, Ibadan, Nigeria; ^5^Institute for Global Health, University College London, London, United Kingdom; ^6^Save the Children International, Abuja, Nigeria; ^7^Save the Children UK, London, United Kingdom; ^8^GlaxoSmithKline (GSK), Lagos, Nigeria; ^9^Eudowood Division of Pediatric Respiratory Sciences, Department of Pediatrics, School of Medicine, Johns Hopkins University, Baltimore, MD, United States; ^10^Department of Paediatrics, University College Hospital, Ibadan, Nigeria; ^11^Centre for International Child Health, Murdoch Children’s Research Institute, University of Melbourne, Royal Children’s Hospital, Parkville, VIC, Australia

**Keywords:** knowledge, care seeking, acute respiratory infection, pneumonia, sub-Saharan Africa

## Abstract

**Background:**

Between 2013 and 2022, Nigeria did not meet globally defined targets for pneumonia control, despite some scale-up of vaccinations, oxygen and antibiotics. A deliberate focus on community-based programs is needed to improve coverage of protective, preventive and treatment interventions. We therefore aimed to describe caregiver knowledge and care seeking behaviour for childhood pneumonia, in a high child mortality setting in Nigeria, to inform the development of effective community-based interventions for pneumonia control.

**Methods:**

We conducted a cross-sectional household survey in Kiyawa Local Government Area, Jigawa State, Nigeria between December 2019 and March 2020. We asked caregivers about their knowledge of pneumonia symptoms, prevention, risks, and treatment. A score of 1 was assigned for each correct response. We showed them videos of pneumonia specific symptoms and asked (1) if their child had any respiratory symptoms in the 2-weeks prior; (2) their subsequent care-seeking behaviour. Multivariate regressions explored socio-demographic and clinical factors associated with care seeking.

**Results:**

We surveyed 1,661 eligible women, with 2,828 children under-five. Only 4.9% of women could name both cough and difficulty/fast breathing as pneumonia symptoms, and the composite knowledge scores for pneumonia prevention, risks and treatment were low. Overall, 19.0% (536/2828) of children had a report of pneumonia specific symptoms in the prior two-weeks, and of these 32.3% (176/536) were taken for care. The odds of care seeking was higher among children: with fever (AOR:2:45 [95% CI: 1.38–4.34]); from wealthiest homes (AOR: 2:13 [95% CI: 1.03–4.38]) and whose mother first married at 20–26 years compared to 15–19 years (AOR: 5.15 [95% CI: 1.38–19.26]). Notably, the caregiver’s knowledge of pneumonia was not associated with care seeking.

**Conclusion:**

While some socio-demographic factors were associated with care seeking for children with symptoms of Acute Respiratory Infection (ARI), caregiver’s knowledge of the disease was not. Therefore, when designing public health interventions to address child mortality, information-giving alone is likely to be insufficient.

## Introduction

Global progress in pneumonia control has been uneven, despite the considerable achievement in reducing global under-five mortality from an estimated 93 deaths per 1,000 livebirths in 1990, to 39 deaths per 1,000 livebirths in 2018 (1). Approximately 90% of global under-five deaths occur in low-and middle-income countries (LMICs), and pneumonia accounts for 18% of these deaths. In 2022, Nigeria had the highest absolute number of childhood pneumonia deaths (2), with the persistent challenge of poor and inequitable coverage of proven interventions such as vaccination, antibiotics, and oxygen therapy (3–7). Nigeria launched its first Pneumonia Control Strategy in 2020, with the aim to reduce childhood morbidity and mortality from pneumonia (8). Community prevention of pneumonia and improvement in care-seeking practices were highlighted as key strategic priorities in this strategy and implementation plan for Nigeria (9). Therefore community-focussed interventions, which are adapted to local contextual needs will be important in the reduction of pneumonia burden in Nigeria.

The 2018 Nigeria Demographic and Health Survey reported that 3% of surveyed children under-five had symptoms of pneumonia and of these, 23% received care from government facilities and 37% received care from a private patent medicine vendor (PPMV) (10). Care seeking varied by geopolitical zones, household wealth index and maternal education (10). Children living in wealthier households, from the South–South geopolitical zone and whose mother had at least secondary education were more likely to be taken for care than those in poorer households, other geopolitical zones, or less educated mothers (10). Previous studies from low- and middle-income countries globally have also identified child sex and age (11, 12), maternal education and marital status (13–15), household dynamics and wealth index (11, 15, 16), perceived illness severity and trust in health care (12) as determinants of care seeking for sick under-five children. However, these relationships can differ by setting, highlighting the importance of local empirical studies on care seeking for children under-five to guide interventions design and prioritization for child health programming (17). Evidence on care seeking for children in Nigeria has focussed mostly on febrile illnesses (17–20). Prior studies on care seeking for pneumonia are either non-empirical (14, 21–24),or hospital based studies (25, 26),with a few focusing on caregiver’s knowledge (27). Nevertheless, this evidence reiterates that for interventions to improve care seeking practices for children and reduce under-five mortality, we need to address the inapparent yet potent contextual factors such as social and gender norms, inequity, economic and health system related factors.

Achieving Sustainable Development Goal target 3.2, to end preventable under-five deaths by 2030, will be difficult if the underlying issue of poor care seeking practice for childhood pneumonia among caregivers is not given due consideration (28, 29). Therefore, we conducted this study to provide context specific understanding of caregiver knowledge and care seeking behaviour for childhood pneumonia, to help inform effective interventions for pneumonia control in Nigeria.

## Materials and methods

We conducted a cross-sectional household survey as part of the baseline phase of the INSPIRING Jigawa community-based cluster randomized controlled trial (ISRCTN 39213655) (30). Data were collected between December 2019 and stopped early on 18^th^ March 2020 due to COVID-19 restrictions, therefore we could not complete data collection in 1 of 33 clusters.

### Setting

This study was conducted in Kiyawa Local Government Area (LGA), Jigawa State, North-Western Nigeria. According to 2021 Multiple Indicators Cluster Survey (MICS), Jigawa State had the second highest under-five mortality rate in Nigeria (174 deaths per 1,000 live births), and is not on track to meet global SDG 3.2 target until the year 2071–41 years after the target date (29, 31). Jigawa is majority rural with 85% of its people being farmers, and 50% of the population in the poorest wealth quintile for Nigeria (10). Kiyawa LGA, one of 27 LGAs, has 11 administrative wards with an estimated population of 250,000 and 33 primary government health facilities and was purposively selected for the study.

### Population and sampling

For this analysis, we focussed on women, aged 16–49 years, with a child under the age of 5 years at the time of the survey and who is normally resident in the compound. The full sampling procedure has been described elsewhere (30). Briefly, we used a three-step systematic random sampling approach to select eligible women using the following steps: 1.) Community mapping to identify the total number of compounds in each community and label each compound with a unique sequential number. 2.) Use of random number generator to determine the first compound for recruitment, with a standard sampling interval of 10 applied thereafter. In every community we sampled a minimum of 3 compounds, meaning in some smaller communities we sampled more than one in every ten compounds. 3.) In each selected compound, the compound head was interviewed to obtain information on compound composition, including the number of women with under-five children living in the compound. A unique identifier was assigned to each of the women and a random number generator (embedded within the electronic data collection tool) informed data collectors which woman should be interviewed. In cases where the head of the compound did not give consent to take part in the study, there was no eligible women resident in the compound, or all those who are normally resident were not around at the time of visit and were not expected to return within 1–2 days, the next compound was sampled. All villages in the LGA were included.

### Data collection

The data was collected through interviewer-administered surveys by trained teams of data collectors, who had at least secondary education and proficiency in the local dialect (Hausa language). They were trained for one-week, with 3 days of in-class teaching on study protocol, research tools, and interview techniques, and another 3 days of field practice where the data collectors watched experienced researchers conduct the interview and thereafter simulated the process (32). In each selected compound, they interviewed the compound head and the sampled eligible woman. We obtained sociodemographic information, compound composition, and assets from the heads of compound. We interviewed woman on pneumonia knowledge, recent illness episodes and care seeking for sick children under-five years. The women were shown videos of children with fast breathing, chest in-drawing, noisy breathing and difficulty in breathing. The videos were shown to the women during the survey and were not used as education materials but rather to aid recall of symptoms, given the limitations which have previously been reported in the Demographic Health Survey (which our tool was based on). (33, 34). For each video, they were asked to indicate if their children had similar symptoms in the 2 weeks preceding data collection. Data was collected using CommCare on password protected Android tablets. Five people provided in-the-field supervision to the data collectors to ensure data quality. Periodic data cleaning was done, and errors were discussed among the authors and with the data collectors through one-on-one discussions or monthly review meetings as deemed appropriate by the project managers (JS and DB).

### Analysis

The primary outcomes of interest were caregiver’s knowledge of pneumonia and care-seeking for children with symptoms of Acute Respiratory Infection (ARI). Despite being interested in pneumonia, we are reliant on care-giver reported symptoms, and therefore are classifying any report of cough, fast/difficult breathing or chest-indrawing as an “acute respiratory infection” (ARI), in line with the DHS approach, as an approximation for pneumonia (10).

Knowledge of pneumonia was measured across four domains: symptoms, prevention, risk and treatment. Correct knowledge of pneumonia symptoms was defined as the caregiver reporting both cough and fast/difficulty in breathing; this definition was chosen as it is the entry criteria for conducting a pneumonia presentation in the WHO’s Integrated Management of Childhood Illnesses (IMCI) guidelines. For other domains, a score of 1 was given for each correct response based on the Global Action Plan for Control of Childhood Pneumonia and Diarrhea (see Table 1) (35). We then summed these scores for each domain and generated a composite knowledge score combining prevention, risk, and treatment.

**Table 1:** Knowledge of pneumonia among caregivers of children under-five in Kiyawa LGA, Jigawa state Nigeria.

Variables	Frequency (%) (*N* = 1,661)
*Knowledge of symptoms*	
Cough	672 (40.5)
Rapid or difficult breathing	110 (6.6)
Knowledge of cough AND rapid/difficult breathing	81 (4.9)
*Knowledge of preventive strategies for pneumonia*	
Vaccination	763 (45.9)
Exclusive breastfeeding for 6 months	315 (19.0)
Good nutrition	199 (12.0)
Hand washing	146 (8.8)
*Knowledge of pneumonia transmission risks*	
Breathing dust	344 (20.7)
Poor hygiene	308 (18.5)
Being near someone sick	55 (3.3)
Being near someone coughing	56 (3.4)
*Knowledge of treatments for pneumonia*	
Antibiotics	605 (36.4)
Oxygen therapy	70 (4.2)
*Knowledge scores*	Mean ± SD
Preventive strategies (maximum 4)	0.9 ± 1.1
Transmission risks (maximum 4)	0.5 ± 0.7
Treatment (maximum 2)	0.4 ± 0.5
Total pneumonia knowledge score (maximum 10)	2.0 ± 2.0

Care seeking was defined as a positive response to the question: “Have you sought care for your child outside home in the last 2 weeks?” Prompt care seeking was defined as care sought outside the home within 48 h of the caregiver recognising the child was sick. All data was self-reported by the child’s mother and we were unable to verify these responses with clinical records.

We reported descriptive statistics for respondents’ sociodemographic characteristics, knowledge of pneumonia and care seeking patterns for children under five. We used chi-square and fisher exact tests to assess factors associated with prompt care seeking for children with ARI symptoms. To assess the relationship between knowledge (exposure - using the composite knowledge score) and care seeking for children with ARI symptoms (outcome), we conducted a multivariable logistic regression. We included the following confounders based on *a priori* evidence from literature and contextual knowledge: household wealth index, woman’s age, marital ranking; education, job, previous child loss and bad obstetric history, child age and sex, and reported symptoms during the illness episode. We analyzed the data using STATA version 17.

## Results

We recruited 1,661 eligible women from 2,378 sampled compounds, who provided data on 2,828 children under-five (median 2 child under-five per woman, range: 1–8). A description of the socio-demographic characteristics of the women and compounds included have been previously published (32). In brief, 64.0% of the compound heads had an informal or religious education, with 55.6% of them practicing subsistence farming as their main source of livelihood. Of the women, nearly half (47.0%) were aged 20–29 years, over half (55.1%) had informal or religious education, Over 80% had more than one child and 7.3% had experienced a stillbirth (see Appendices 1, 2).

### Pneumonia knowledge

A total of 672 (40.5%) women identified cough as a pneumonia symptom, but just 4.9% had “correct knowledge” of pneumonia specific symptoms (i.e., naming both cough and difficult/fast breathing) – Table 1. Only 1.0% of women correctly reported all four risk factors for pneumonia, and the most commonly answered risks were not correct, with 52.0% reporting cold weather and 34.9% bathing in cold water as risks. A total of 605 (36.4%) and 70 (4.2%) women knew antibiotics and oxygen therapy, respectively, as treatments for pneumonia. Mean knowledge scores were low for prevention (mean = 0.9 of a maximum 4, standard deviation (SD) ±1.1), transmission (mean = 0.5 of a maximum 4, SD ±0.7) and treatments (mean = 0.4 of a maximum 2, SD ±0.5; Table 1). Nearly half of the respondents did not know any symptoms, preventive strategies, transmission risk factors and treatments for pneumonia (see Appendix 2).

### Care seeking

**Figure 1:**
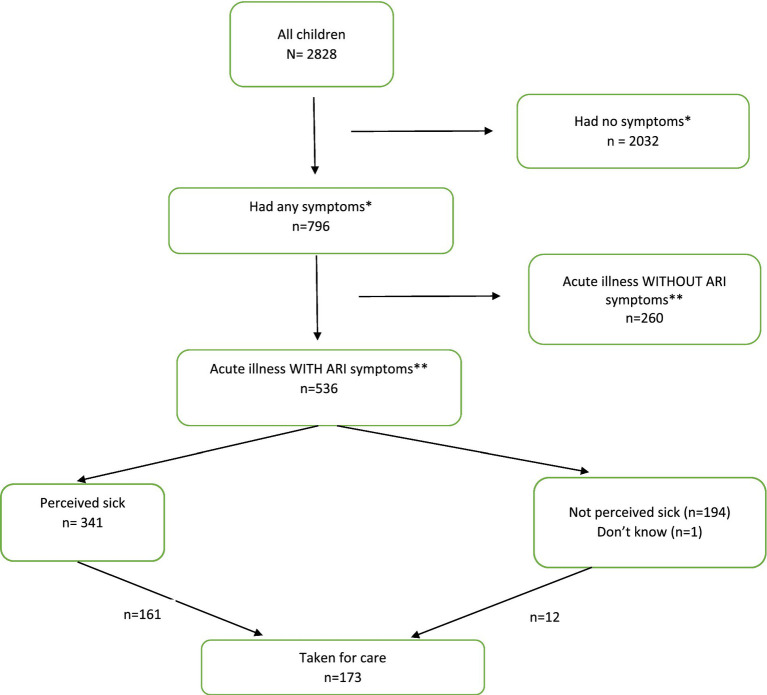
Children inclusion flow diagram.

796/2828 (28.1%) of children had symptoms of acute illness in the prior 2 weeks, including 536 (19.0%) with ‘ARI symptoms’ (i.e., caregiver reported cough, fast/difficult breathing, or lower chest in-drawing). Of those with ARI symptoms, 63.6% (341/536) were considered sick by their mother, and less than half were taken for care (161/341, 47.2%) – Figure 1. Overall, 240 children (8.5%) had been taken for care outside of the home in the prior 2-weeks, and of these 72% (173/240) were taken for pneumonia specific symptoms/signs. A greater proportion of children with pneumonia symptoms were taken for care than those with acute illness without pneumonia symptoms (32.3% versus 25.4%, *p* = 0.194).

Prior to seeking care outside of the home, 8.2 and 8.8% of caregivers reported giving children with pneumonia symptoms antibiotics and antimalarials respectively, and this was 6.9 and 5.8% for children without pneumonia symptoms. Pre-consultation treatment with cough syrup was higher among children with pneumonia symptoms (17.5%) compared to children without pneumonia symptoms (3.1%) (*p* < 0.001). After being taken for care, antibiotics treatment was higher among children with pneumonia symptoms (57.2%) compared with those without pneumonia symptoms (37.2%) (*p* = 0.006) but antimalarial treatment was similar between the groups (27.8 and 25.4%, respectively; Table 2).

**Table 2:** Description of care seeking practices for children with symptoms of acute illness in Kiyawa LGA.

Variables		Acute illness without ARI symptoms (*N* = 260)	Acute illness with ARI Symptoms (*N* = 536)	*p*-value
		Frequency (%)	Frequency (%)	
Taken for care	Yes	66 (25.4)	173 (32.3)	0.121
	No	193 (74.2)	361 (67.3)	
	Do not know/missing	1 (0.4)	2 (0.4)	
		(N = 66)	(N = 173)	
Timeliness of care seeking	<48 h	44 (66.7)	102 (59.0)	0.296
	≥ 48 h	22 (33.3)	70 (40.4)	
	Not sure	–	1 (0.6)	
Number of care seeking episodes	Once	60(90.9)	142 (82.1)	0.126
	More than once	6 (9.1)	29 (16.8)	
	Not sure	–	2 (1.1)	
Home treatment before seeking care	Cough syrup	8 (3.1)	94 (17.5)	**<0.001**
	Antibiotics	18 (6.9)	44 (8.2)	0.575
	Antimalaria	15(5.8)	47 (8.8)	0.139
	Paracetamol	4 (1.5)	12 (2.2)	0.358^a^
	Traditional medicine	2(0.8)	13 (2.4)	0.163^a^
	No treatment	6 (2.3)	12 (2.2)	0.951
First location of care seeking	Family/friend	1 (1.5)	4 (2.3)	0.974^a^
	Traditional healer	2 (3.0)	5 (2.9)	
	(TBA/CHEW)	2 (3.0)	2 (1.2)	
	Pharmacist	4 (6.0)	11 (6.4)	
	PPMV	11 (16.4)	29 (16.9)	
	Primary facility	44 (65.7)	110 (64.0)	
	Private hospitals	3 (4.5)	10 (5.8)	
	Secondary hospital	0 (0.0)	1 (0.6)	
Accompanying caregivers	Father	12(18.1)	22 (12.7)	**<0.001** ^a^
	Mother	48 (72.7)	102 (59.0)	
	Mother and Father	3 (4.5)	47 (27.2)	
	Other family	3 (4.5)	2 (1.1)	
Care seeking decision	Father	40 (60.6)	59(34.1)	**<0.001**
	Mother	18 (27.3)	52 (30.1)	
	Mother and Father	8 (12.1)	59 (34.1)	
	Other family^c^	0 (0.0)	3 (1.7)	
Cost of care (N)^d^	Median (range)	850 (200–4,000)	1,000 (100–32,000)	**0.020** ^b^
Treatments received after seeking care	Antibiotics	25(37.2)	99 (57.2)	**0.006**
	Paracetamol	35 (52.2)	92 (53.2)	0.896
	Antimalaria	17 (25.4)	48 (27.8)	0.711
	Traditional medicine	1(1.5)	11(6.4)	0.187^a^
Decline treatment	No	63(95.5)	155 (89.6)	0.566^a^
	Yes	3(4.6)	13 (7.5)	
	Not sure	0 (0.0)	5 (2.9)	
Household wealth index	Lowest	8 (11.9)	27 (15.6)	0.233
	Low/Middle	9 (13.4)	32 (18.5)	
	Middle	17 (25.4)	25 (14.5)	
	Middle/High	11 (16.4)	39 (22.5)	
	Highest	22 (32.8)	50 (28.9)	

a Fisher exact test.

b Mann–Whitney U test.

c Other family includes grandparents, aunt or uncle.

d Implausible responses (*n* = 41) were set to missing.Bold values indicate statistical significance.

**Figure 2:**
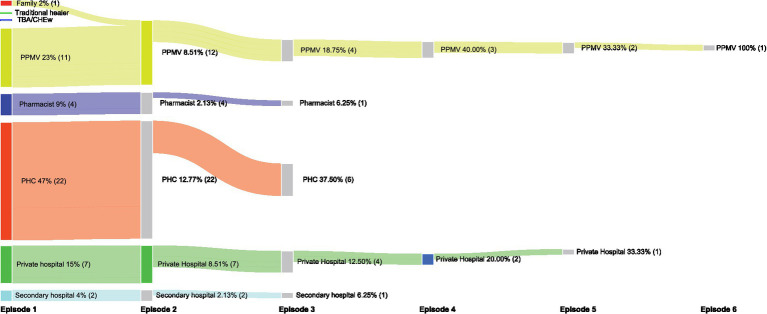
Care seeking pattern for children with ARI symptoms.

Seeking care from multiple providers was common for children with ARI symptoms, with 17% going to more than one place (range 1–5). The most common place to seek care was public primary health facilities, followed by PPMVs. Caregivers mostly went to the same places when seeking care multiple times (Figure 2). The cost of treatment was higher for children with ARI symptoms compared to non-ARI symptoms cases (*p* = 0.020) and they were more likely to be taken for care by both parents (*p* < 0.001; Table 2).

### Relationship between knowledge and care seeking

Table 3 presents the adjusted logistic regression model, exploring the relationship between knowledge and care seeking. Maternal pneumonia knowledge, along with marital ranking among husband’s wives, maternal age, previous child loss and child sex were not associated with care seeking. The adjusted odds (aOR) of care seeking was higher among children with fever compared to those without fever (aOR:2:45 [95% CI: 1.38–4.34]), children whose mother first married when they were older - aged of 20–35 years versus mothers married at ages 10–14 years (AOR: 5.15 [95% CI: 1.38–19.26]), and children from the wealthiest homes compared to the poorest homes (AOR: 2:13 [95% CI: 1.03–4.38]). However, children whose mothers were not subsistence farmers had lower odds of care-seeking.

**Table 3:** Result of multivariate analysis of factors associated with care seeking for children with ARI.

symptoms						
Variables	Crude analysis			Adjusted analysis		
	OR	95% CI	*p*-value	aOR	95% CI	*p*-value
*Total knowledge score*	0.97	0.83–1.10	0.604	1.08	0.94–1.24	0.287
*Woman marital ranking*						
Highest rank amongst wives	1.00			1.00		
Lower rank amongst wives	1.14	0.70–1.84	0.602	1.03	0.61–1.74	0.910
*Woman age*						
16–20 years	1.00			1.00		
20–29 years	0.76	0.29–1.99	0.575	0.84	0.31–2.31	0.740
30–39 years	0.80	0.30–2.15	0.658	0.91	0.32–2.57	0.856
40–49 years	0.98	0.32–3.00	0.966	1.12	0.34–3.73	0.852
*Woman education*						
No education	1.00			1.00		
Primary/Informal	1.88	1.06–3.32	**0.030**	1.32	0.70–2.48	0.388
Secondary and above	1.88	0.67–5.27	0.228	1.12	0.37–3.45	0.834
*Woman job*						
Subsistence farmer	1.00			1.00		
Unskilled manual labor	0.41	0.15–1.08	0.072	0.29	0.10–0.83	**0.022**
Skilled manual labor	0.53	0.20–1.43	0.213	0.49	0.17–1.44	0.126
Small business owner/professional	0.42	0.17–0.97	**0.042**	0.37	0.14–0.95	**0.038**
Not working	0.23	0.07–0.76	**0.016**	0.23	0.07–0.83	**0.025**
*Child’s age (month)*						
0–11 months	1.00			1.00		
12–23 months	1.08	0.59–1.99	0.796	1.04	0.53–2.00	0.916
25–59 months	1.17	0.68–2.00	0.560	1.34	0.75–2.38	0.320
Child sex						
Female	1.00			1.00		
Male	0.97	0.65–1.45	0.890	0.97	0.63–1.51	0.908
*Household wealth index*						
Lowest socioeconomic	1.00			1.00		
Low/Middle	1.75	0.87–3.53	0.118	2.14	1.00–4.58	0.050
Middle	1.55	0.76–3.19	0.228	1.33	0.62–2.84	0.468
Middle/High	2.49	1.28–4.84	**0.007**	2.68	1.31–5.49	**0.007**
Highest	1.75	0.90–3.39	0.095	2.13	1.03–4.38	**0.040**
*Symptoms*						
Fever	3.66	2.15–6.22	**<0.001**	2.45	1.38–4.34	**0.002**
Diarrhea	1.63	0.97–2.77	0.067	1.27	0.72–2.24	0.418
Cough	1.15	0.35–3.69	0.814	0.84	0.22–3.23	0.809
Breathing issues^a^	1.24	0.51–2.06	0.463	1.09	0.59–2.00	0.791
Chest indrawing	1.03	0.51–2.06	0.938	1.18	0.56–2.48	0.662
*Experience of child loss* ^b^						
No	1.00			1.00		
Yes	1.35	0.75–2.41	0.317	1.65	0.87–3.13	0.124
Age at first marriage						
10–14 years	1.00			1.00		
15–19 years	1.19	0.71–2.00	0.496	1.52	0.86–2.67	0.147
20–35 years	4.58	1.44–14.47	**0.010**	5.15	1.38–19.26	**0.015**

a Fast breathing, difficulty in breathing or noisy breathing.

b Child death or stillbirth.Bold values indicate statistical significance.

An exploratory analysis of timely care-seeking as the outcome found similar associations with children who had fever (*p* = 0.004), were from wealthy home (*p* = 0.010) or whose mother had higher levels of education than informal or religious education (*p* = 0.021) or got married at an older age (*p* = 0.002) had higher odds of prompt care. In contrast, delayed care seeking was higher among children whose mother had previous child loss (*p* = 0.001) (Appendix 3). A logistic regression was not conducted due to the small number of cases.

## Discussion

This study aimed to provide context specific understanding of caregiver knowledge and care seeking behaviour for children with pneumonia specific symptoms. We found that nearly 1 in 5 children had symptoms that could indicate a pneumonia infection within the prior 2 weeks, and care seeking for these children was low. Caregiver’s knowledge of pneumonia was also low and not associated with care seeking behaviour. The caregiver reporting the child having a fever was associated with timely care seeking, while prior child loss was associated with a non-significant increase in care-seeking, but this care-seeking was more commonly delayed. These findings suggest that care seeking for children under-five in this setting may be driven by lived experiences and community norms about childhood illness; hence, community-based interventions aimed at improving care seeking must not focus only on improving caregiver’s knowledge but address wider contextual factors.

The first step in the quest to seek care is recognising that the child is sick, and that the illnesses are serious enough to require clinical examination and potentially treatment. In this setting we observed that 36% of children with reported ARI symptoms were not considered to be “sick.” Possible explanations of this could be that that their symptoms were perceived as not serious or the normative care seeking practices in the setting do not include seeking care outside home. We had reflected on this finding with our research team and a common report was caregivers attributing childhood illness to teething. While they reported that many caregivers believed teething would resolve spontaneously, we also observed caregivers attributing child death to teething in this setting (36, 37). In our study, fever was associated with both care seeking and timely care seeking practices. This likely reflects a maternal perception of fever as being a serious symptom which requires urgent medical attention, as reported in other studies (38–40). The level of pneumonia knowledge was low among the caregivers. While studies have reported low level of pneumonia knowledge among caregivers in similar settings in Nigeria (41, 42),we found more than 40% of the caregivers could not name any symptoms, treatments, prevention strategies or risk factors for pneumonia and among those who provided response, more than 50% mentioned cold weather or bathing with cold water as key risk factors for pneumonia. Concerning was less than 5% of women knowing that being close to someone sick or coughing are risk factors. Addressing this misinformation and gaps in knowledge about pneumonia risks requires urgent attention to increase community awareness about the burden of pneumonia. This may help to reduce pneumonia incidence because caregivers will become empowered to take appropriate steps to prevent childhood pneumonia. In Nigeria, caregivers dress children in thick clothing during cold weather to prevent pneumonia rather that addressing issues of overcrowding, indoor air pollution and poor hygiene which increase the risk of respiratory infection (42, 43).

Approximately 1 out of every 5 children with ARI symptoms received cough syrup before care was sought. While this is not surprising given that 28% of the caregivers identified cough syrup as a treatment option for pneumonia, it however underscores need for more public awareness about the risks associated with use of cough syrups among under-five children such as convulsion rapid heart rate and death (44). Our study also points to possible overuse of antibiotics. In Nigeria, the leading causes of morbidities and mortalities among children under-five are pneumonia, malaria and diarhoea (45, 46),with antibiotics only indicated for pneumonia. We found that more than two-thirds of children with no pneumonia symptoms were prescribed antibiotics. Given that the majority of children received care from primary health facilities and PPMVs, campaigns against antibiotics misuse need to include lower-level providers and community engagement.

The poor knowledge of medical oxygen as part of treatment for children with pneumonia among the respondents may in part explain caregiver’s refusal of medical oxygen for treatment of children with pneumonia in this setting (47). Fear of medical oxygen has been reported in similar settings within and outside Nigeria (47, 48). Considering the increased investment in medical oxygen due to the COVID-19 pandemic, it is important that communities are informed about the importance of medical oxygen so that acceptance of oxygen is high and demand for this service is established.

In contrast to previous studies which have reported associations between a child’s sex and care seeking practice (12, 49, 50), we did not observe such an association in our study. This may be explained by increased advocacy about gender equality in the setting and eroding sex preference practices. Another possible explanation may be increased male involvement in care seeking decision making and practices for children with pneumonia symptoms. This agrees with a study conducted by Dougherty et al. in Nigeria, which found that fathers take their children for care when the mother is not available or accompany the mother to health facilities when their child is sick (16). Fathers’ involvement in care seeking practices eliminates potential socio-cultural and economic factors that may deter care seeking actions. Our finding of no association between care seeking and a child’s age, however, may require further exploration.

We found evidence of a possible effect of health literacy on care seeking behaviour (51). For example, women with a higher level of education, who married at adult age or lived in wealthy households more often took their children for care. Interestingly, women with previous child loss delayed care seeking for their children. This may suggest lack of trust in healthcare based on previous negative outcomes, but may also highlight persisting barriers such as socio-cultural issues, geographical access to quality health services, and health literacy. It will be important to explore the barriers faced in accessing care for this group of caregivers.

Our study had two key limitations. Firstly, we relied on mother’s recall ability and could not confirm for all children the presence of pneumonia symptoms and the timeliness and pattern of care seeking. Given the cross-sectional nature of the study, we could not account for seasonal variation in prevalence of pneumonia symptoms and impacts on care seeking. Secondly, we did not explore how household power dynamics and sociocultural factors might have influenced care seeking decisions. Nevertheless, given the sampling approach findings from this study should be generalizable to other settings within and outside Nigeria with similar sociocultural beliefs and health system structures. Finally, while not a limitation, it is important to note that the low level of pneumonia knowledge among caregivers may not represent the current situation given the public health messaging around preventing transmission of COVID-19.

## Conclusion

Care seeking for children under-five who had ARI symptoms was not associated with caregiver’s knowledge of the disease. Lived experiences and community perceptions of childhood illnesses may play a more important role in this setting. Therefore, when designing public health interventions to address child mortality, information-giving alone, is likely to be insufficient. Instead working to challenge pervasive misconceptions and social norms will be needed to ensure that practical barriers to care are minimized.

## Data availability statement

The original contributions presented in the study are included in the article/Supplementary material, further inquiries can be directed to the corresponding author.

## Ethics statement

The studies involving human participants were reviewed and approved by Jigawa State Government (ref: JPHCDA/ADM/GEN/073/ V.I) and the University College London Research Ethics Committee (ref: 3433/004). Written informed consent for participation was not required for this study in accordance with the national legislation and the institutional requirements.

## Author contributions

CK, RB, AB, TC, HG, and AF: study conception. TC, CK, RB, PV, AdI, TO, TA, AB, HG, EM, and AF: study design. DB, JS, OU, and AB: data collection. AB, CK, and HG: data analysis. AB and CK: writing original draft. HG, TC, EM, TA, PV, TO, AdI, AgI, and OU: writing—review and editing. AF, CK, and TC: grant holders. All authors contributed to the article and approved the submitted version.

## Full list of INSPIRING Consortium members

Carina King (Karolinska); Tim Colbourn, Rochelle Ann Burgess, Agnese Iuliano (UCL); Hamish R Graham (Melbourne); Eric D McCollum (Johns Hopkins); Tahlil Ahmed, Samy Ahmar, Christine Cassar, Paula Valentine (Save the Children UK); Adamu Isah, Adams Osebi, Ibrahim Haruna, Abdullahi Magama, Ibrahim Seriki (Save the Children Nigeria); Temitayo Folorunso Olowookere (GSK Nigeria); Matt McCalla (GSK UK); Adegoke G Falade, Ayobami Adebayo Bakare, Obioma Uchendu, Julius Salako, Funmilayo Shittu, Damola Bakare, Omotayo Olojede, Abiodun Sogbesan (University of Ibadan); James Beard (independent consultant).

## Funding

This work was funded through the GlaxoSmithKline (GSK)-Save the Children Partnership (grant reference: 82603743) awarded to TC, CK, and AF. Employees of both funding partners (GSK and Save the Children) contributed to the design and oversight of the wider study as part of our co-design process. Any views or opinions presented are solely those of the author/publisher and do not necessarily represent those of Save the Children or GSK, unless otherwise specifically stated.

## Conflict of interest

TO was employed by GlaxoSmithKline (GSK), Lagos, Nigeria.

The remaining authors declare that the research was conducted in the absence of any commercial or financial relationships that could be construed as a potential conflict of interest.

## Publisher’s note

All claims expressed in this article are solely those of the authors and do not necessarily represent those of their affiliated organizations, or those of the publisher, the editors and the reviewers. Any product that may be evaluated in this article, or claim that may be made by its manufacturer, is not guaranteed or endorsed by the publisher.
