# Supplementary material for: Pneumonia knowledge and care seeking behavior for children under-five years in Jigawa, Northwest Nigeria: a cross-sectional study
Source: Front Public Health. 2023 Jul 18;11:1198225. doi: 10.3389/fpubh.2023.1198225 (PMC10393027; doi:10.3389/fpubh.2023.1198225)
Supplement: Supplementary file 1 [file Table_1.DOCX]

Appendix 1 Participant inclusion flow diagram^[[1]](#footnote-1)^

Appendix 2 Respondents Characteristics

| Attributes | Frequency | Percentage |
| --- | --- | --- |
| **Woman’s age** |  |  |
| 16-19 | 100 | 6.0 |
| 20-29 | 781 | 47.0 |
| 30-39 | 620 | 37.3 |
| 40 -49 | 160 | 9.7 |
| **Level of education*** |  |  |
| No formal education | 521 | 31.5 |
| Informal/religious education | 910 | 55.1 |
| Primary | 129 | 7.8 |
| Secondary | 80 | 4.8 |
| Tertiary/further | 12 | 0.7 |
| **Marital status **** |  |  |
| Married | 1651 | 99.7 |
| Divorced/separated | 3 | 0.2 |
| Widowed | 6 | 0.1 |
| **Occupation** |  |  |
| Subsistence farmer | 84 | 5.1 |
| Unskilled manual labour | 343 | 20.7 |
| Skilled manual labour | 249 | 15.0 |
| Small business owner | 792 | 47.7 |
| Professional | 3 | 0.2 |
| TBA | 1 | 0.1 |
| Not working | 189 | 11.4 |
| **Surviving children (under-five)***** |  |  |
| 1 | 783 | 47.1 |
| 2 | 751 | 45.2 |
| 3 and 4 | 121 | 7.3 |
| **Previous pregnancy outcome** |  |  |
| Livebirth | 1437 | 86.5 |
| Stillbirth | 121 | 7.3 |
| Miscarriage | 101 | 6.1 |
| Abortion | 2 | 0.1 |
| **Woman’s ranking amongst her husband wives****** |  |  |
| 1 | 1311 | 79.6 |
| 2-4 | 335 | 20.4 |
| Age at first marriage***** |  |  |
| 10-14 years | 539 | 32.6 |
| 15-19 years | 1073 | 64.9 |
| 20-26 years | 42 | 2.5 |
| **Wealth quintile** |  |  |
| Lowest | 355 | 31.4 |
| Low/Middle | 366 | 22.0 |
| Middle | 295 | 17.8 |
| Middle/High | 320 | 19.3 |
| Highest | 325 | 19.6 |

* Values recorded as don’t know were set to missing (n=9)

** Don’t know (n=1)

*** *More than 4 set as missing (n=15)

*** Women reporting more than four under-five children were set as missing (n=6)

***** invalid response set as missing (n=7)

Appendix 3 Knowledge of pneumonia among caregivers of under-five children in Kiyawa LGA, Jigawa State Nigeria

| Variables | Frequency | Percentage |
| --- | --- | --- |
| **Knowledge on symptoms** |  |  |
| Cough | 672 | 40.5 |
| Rapid or difficult breathing | 110 | 6.6 |
| Fever | 736 | 44.3 |
| headache | 191 | 11.5 |
| Wheezing | 154 | 9.3 |
| Vomiting | 213 | 12.8 |
| Diarrhea | 213 | 12.8 |
| Death | 18 | 1.1 |
| Don’t know | 738 | 44.4 |
| **Knowledge on prevention** |  |  |
| Vaccination | 763 | 45.9 |
| Avoid open defaecation | 430 | 25.9 |
| Keep house surrounding clean | 379 | 22.8 |
| Exclusive breastfeeding | 315 | 19.0 |
| Safely store household drinking water | 179 | 10.8 |
| Don’t drink dirty water | 251 | 15.1 |
| Avoid contaminated food | 116 | 6.9 |
| Good nutrition | 199 | 12.0 |
| Hand washing | 146 | 8.8 |
| Good sanitation | 121 | 7.3 |
| Use of herbs | 18 | 1.1 |
| Don’t know | 747 | 45.0 |
|  |  |  |
| **Knowledge on transmission risk** |  |  |
| Being cold | 863 | 52.0 |
| Bathing in cold water | 579 | 34.9 |
| Breathing dust | 344 | 20.7 |
| Being bewitched/cursed | 49 | 3.0 |
| Poor hygiene | 308 | 18.5 |
| Being near someone sick | 55 | 3.3 |
| Being near someone coughing | 56 | 3.4 |
| Being weak | 33 | 2.0 |
| No response | 736 | 44.3 |
| **Knowledge on treatment** |  |  |
| Antibiotics | 563 | 33.9 |
| Injectable antibiotics | 181 | 10.9 |
| Antipyretics | 326 | 19.63 |
| Antimalaria | 77 | 4.6 |
| Inhaler | 23 | 1.4 |
| ORS | 35 | 2.1 |
| Vaccination | 355 | 21.4 |
| Oxygen therapy | 70 | 4.2 |
| Cough syrup | 472 | 28.4 |
| Traditional medicine | 41 | 2.5 |
| No response | 772 | 46.5 |

| Variables | Delayed | Timely | P-value |
| --- | --- | --- | --- |
|  | Frequency (%) | Frequency (%) |  |
| **Woman education** |  |  |  |
| No formal education | 23 (41.1) | 33 (58.9) | **0.021*** |
| Informal/Religious | 41 (48.8) | 43 (51.2) |  |
| Primary | 5 (25.0) | 15 (75.0) |  |
| Secondary | 1 (8.3) | 11 (91.7) |  |
| **Age at first marriage** |  |  |  |
| 10-14 years | 36 (56.3) | 28 (43.7) | **0.002*** |
| 15-19 years | 33 (33.7) | 65 (66.3) |  |
| 20—35 years | 1 (10.0) | 9 (90.0) |  |
| **Wealth quintiles** |  |  |  |
| Lowest | 16 (61.5) | 10 (38.5) |  |
| Low/Middle | 13 (40.6) | 19 (59.4) | **0.010** |
| Middle | 13 (52.0) | 12 (48.0) |  |
| Middle/High | 17 (43.60 | 22 (56.4) |  |
| Highest | 11 (22.0) | 39 (78.0) |  |
| **Child sex** |  |  |  |
| Female | 33 (37.9) | 54 (62.1) | 0.455 |
| Male | 37 (43.5) | 48 (56.5) |  |
| **Wife ranking** |  |  |  |
| First | 14 (34.1) | 27 (65.9) | 0.294 |
| Not first | 56 (43.4) | 73 (56.6) |  |
| **Woman occupation** |  |  |  |
| Subsistence farmer | 10 (71.4) | 4 (28.6) | 0.102* |
| Unskilled manual labour | 12 (35.3) | 22 (64.7) |  |
| Skilled manual labour | 7 (28.0) | 18 (72.0) |  |
| Small business owner/Professional | 37 (40.7) | 54 (59.3) |  |
| Not working | 4 (50.0) | 4 (50.0) |  |
| **Woman age** |  |  |  |
| 16-19 years | 3 (33.3) | 6 (66.7) | 0.810 |
| 20-29 years | 32 (40.0) | 48 (60.0) |  |
| **30-39 years** | 29 (39.7) | 38 (60.3) |  |
| 40-49 years | 10 (50.0) | 10 (50.0) |  |
| **Previous child loss** |  |  |  |
| No | 47 (34.3) | 90 (65.7) | **0.001** |
| Yes | 23 (65.7) | 12 (34.3) |  |
| **Breathing issues** |  |  |  |
| No | 54 (44.6) | 67(55.4) |  |
| Yes | 16 (31.4) | 35 (68.6) | 0.106 |
| **Fever/chills** |  |  |  |
| No | 47 (50.5) | 46 (49.5) | **0.004** |
| Yes | 23 (23.1) | 56 (70.9) |  |

Appendix 4 Factors associated with early care seeking practices for children with pneumonia specific symptoms/signs

1. Salako, J., Bakare, D., Colbourn, T., Isah, A., Adams, O., Shittu, F., Uchendu, O., Bakare, A. A., Graham, H., McCollum, E. D., Falade, A. G., Consortium, the I., Burgess, R. A., & King, C. (2023). Maternal mental well-being and recent child illnesses–A cross-sectional survey analysis from Jigawa State, Nigeria. *PLOS Global Public Health*, *3*(3), e0001462. https://doi.org/10.1371/JOURNAL.PGPH.0001462 [↑](#footnote-ref-1)
